# Src Inhibitors Pyrazolo[3,4-d]pyrimidines, Si306 and Pro-Si306, Inhibit Focal Adhesion Kinase and Suppress Human Glioblastoma Invasion In Vitro and In Vivo

**DOI:** 10.3390/cancers12061570

**Published:** 2020-06-14

**Authors:** Marija Nešović, Aleksandra Divac Rankov, Ana Podolski-Renić, Igor Nikolić, Goran Tasić, Arianna Mancini, Silvia Schenone, Milica Pešić, Jelena Dinić

**Affiliations:** 1 Department of Neurobiology, Institute for Biological Research “Siniša Stanković” - National Institute of Republic of Serbia, University of Belgrade, Bulevar Despota Stefana 142, 11060 Belgrade, Serbia; marija.stepanovic@ibiss.bg.ac.rs (M.N.); ana.podolski@ibiss.bg.ac.rs (A.P.-R.); 2 Institute of Molecular Genetics and Genetic Engineering (IMGGE), University of Belgrade, Vojvode Stepe 444a, 11042 Belgrade, Serbia; aleksandradivac@imgge.bg.ac.rs; 3 Clinic for Neurosurgery, Clinical Center of Serbia, Pasterova 2, 11000 Belgrade, Serbia; i.m.nikolic@gmail.com (I.N.); dr.gorantasic@gmail.com (G.T.); 4 School of Medicine, University of Belgrade, Doktora Subotića 8, 11000 Belgrade, Serbia; 5 Department of Pharmacy, University of Pisa, Via Bonanno 6, 56126 Pisa, Italy; arianna.mancini3@gmail.com; 6 Department of Biotechnology, Chemistry and Pharmacy, University of Siena, Via A. Moro 2, I-53100 Siena, Italy; 7 Department of Pharmacy, University of Genova, Viale Benedetto XV 3, 16132 Genova, Italy; schenone@difar.unige.it

**Keywords:** glioblastoma, Src tyrosine kinase inhibitor, cancer invasion, focal adhesion kinase, matrix metalloproteinase

## Abstract

Glioblastoma (GBM), as the most aggressive brain tumor, displays a high expression of Src tyrosine kinase, which is involved in the survival, migration, and invasiveness of tumor cells. Thus, Src emerged as a potential target for GBM therapy. The effects of Src inhibitors pyrazolo[3,4-*d*]pyrimidines, Si306 and its prodrug pro-Si306 were investigated in human GBM cell lines (U87 and U87-TxR) and three primary GBM cell cultures. Primary GBM cells were more resistant to Si306 and pro-Si306 according to the 3-(4,5-Dimethyl-2-thiazolyl)-2,5-diphenyl-2H-tetrazolium bromide (MTT) assay. However, the ability of all GBM cells to degrade the extracellular matrix was considerably compromised after Si306 and pro-Si306 applications. Besides reducing the phosphorylation of Src and its downstream signaling pathway components, both compounds decreased the phosphorylated form of focal adhesion kinase (FAK) and epidermal growth factor receptor (EGFR) expression, showing the potential to suppress the aggressiveness of GBM. In vivo, Si306 and pro-Si306 displayed an anti-invasive effect against U87 xenografts in the zebrafish embryo model. Considering that Si306 and pro-Si306 are able to cross the blood–brain barrier and suppress the spread of GBM cells, we anticipate their clinical testing in the near future. Moreover, the prodrug showed similar efficacy to the drug, implying the rationality of its use in clinical settings.

## 1. Introduction

Glioblastoma (GBM) is the most frequent and aggressive primary brain tumor, classified by the World Health Organization (WHO) as grade IV [1,2]. GBM is characterized by genetic mutations, morphological alterations, proliferation, invasiveness and migration of tumor cells which all lead to the development of diffuse and infiltrative tumors that cannot be completely surgically removed [3,4]. The invasion and migration of GBM cells into normal brain tissue is among the main reasons for unsuccessful GBM treatment [5,6]. As GBM tumors are very aggressive [7,8], the survival rate of GBM patients is generally less than one year [9]. The current standard treatment for GBM patients is surgical resection followed by concomitant radiotherapy with temozolomide chemotherapy [7,10]. Nevertheless, even with current treatment against GBM, lethal relapses usually occur with more aggressive and resistant behavior [9]. A relapse is typically connected with resistant phenotype of the tumor and necessitates new therapeutic approaches for GBM treatment. Importantly, GBM relapses at places distant from its primary site, implying that its cells are spread throughout the brain parenchyma before surgical resection of the tumor is performed [11]. Therefore, it is reasonable to consider neoadjuvant therapy which would suppress the GBM invasion before surgery. This type of approach is common for other malignancies such as lung carcinoma [12].

Altered signaling pathways that lead to GBM pathogenesis represent potential therapeutic targets [13]. Changes commonly occur in signaling, mediated by growth factors and their receptors: the endothelial growth factor, vascular endothelial growth factor, and platelet-derived growth factor. Alterations also occur in kinases including tyrosine-protein kinase Met, phosphatidylinositol-3-kinase (PI3K), and Src-family kinases (SFKs) [3]. Considering that SFKs participate and communicate with the majority of signaling pathways involved in tumor proliferation, cell–cell or cell–extracellular matrix (ECM) interaction, motility, invasiveness and neoangiogenesis, they have emerged as key targets for cancer treatment [14].

The Src tyrosine kinase, also known as c-Src, is the best described member of SFKs, and its overexpression plays a crucial role in GBM pathogenesis [3,15]. The increased Src activity in GBM tumors is also a consequence of the increased activation of proteins upstream of Src (e.g., focal adhesion kinase—FAK), cell surface growth factor receptors (e.g., epidermal growth factor receptor—EGFR), and integrins [7,16]. EGFR has been extensively studied for decades as a clinical marker in GBM and other gliomas [17]. The EGFR gene is the most commonly amplified gene in GBM [18] and these tumors often carry constitutively active EGFR variants [19]. FAK, a non-receptor tyrosine kinase, regulates signal transduction in integrin-enriched focal adhesions enabling communication between the cell and the extracellular matrix [20]. FAK acts directly on the actin cytoskeleton via the tyrosine phosphorylation of key actin-associated proteins, but it can also regulate gene transcription, altering the ability of cells to migrate and invade. Cancer cells with high metastatic potential are characterized by increased activity of markers involved in adhesion, invasion, and migration such as FAK and its downstream signaling pathway members [21]. Src activation, via its downstream member PI3K, leads to the activation of protein kinase B (AKT) and consequently promotes the survival and growth of tumor cells [22]. Through RAS/mitogen-activated protein kinase (MAPK), Src activates extracellular signal-regulated kinase (ERK), and its activated form pERK further leads to cell cycle progression, cell growth and proliferation [23].

Small molecule compounds have previously been selected as good candidates for SFK inhibition, among which dasatinib was the most prominent candidate. However, a clinical evaluation showed that the success of dasatinib in GBM treatment had limitations due to its poor pharmacokinetic profile [24,25]. 

Novel small molecule compounds with a pyrazolo[3,4-d]pyrimidine scaffold, Si306 and its prodrug pro-Si306, are ATP-competitive tyrosine kinase inhibitors and can be used to inhibit SFKs [26,27]. A number of pyrazolo[3,4-d]pyrimidines already showed considerable anti-cancer activity in vitro against a number of GBM [14,28,29] and other cancer cell lines [30,31,32,33,34,35,36,37,38,39], as well as in vivo [35,39,40]. We previously reported that Si306 in combination with radiotherapy significantly inhibited the growth of human GBM U87 cell line xenografts in nude mice compared to the control and single treatment [40]. Our recent study showed that Si306 induced apoptotic death in patient-derived cell lines from the invasive region and core of GBM [41]. Both Src tyrosine kinase inhibitors (STKIs), Si306 and its prodrug, were shown to successfully pass the blood–brain barrier (BBB) [40]. Moreover, pro-Si306 showed enhanced solubility and efficacy in an orthotopic model of GBM compared to Si306. Besides their ability to pass the BBB, Si306 and pro-Si306 successfully, in a dose-dependent manner, inhibited the function of P-glycoprotein (P-gp), an ATP-binding cassette (ABC) transporter member highly expressed in multidrug resistant (MDR) cancer cells [28]. As a result, these compounds showed the ability to reverse paclitaxel resistance in MDR glioblastoma U87-TxR cells [28]. However, due to better solubility, the slow release of the drug and overall prolonged exposure of the brain, pro-Si306 can have advantages over Si306 in clinical trials [28]. 

In this study, we investigated the anti-invasive potential of Si306 and pro-Si306 in GBM cells in vitro and in vivo. The activity of pyrazolo[3,4-d]pyrimidine derivatives was evaluated in human GBM cell lines U87 and U87-TxR, as well as primary GBM cultures established from fresh human tissue samples. In addition, the anti-invasive effect of Si306 and its prodrug were evaluated in vivo in a zebrafish embryo U87 xenograft model. Due to the fact that prodrugs often have considerably lower biological activity compared to their corresponding drugs [42], our aim was to compare the anti-invasive effects between Si306 and pro-Si306 and show that pro-Si306 could be considered as a valuable anti-glioblastoma agent with favorable pharmacokinetic profile.

## 2. Results

### 2.1. Sensitivity of GBM Cells to Si306 and Pro-Si306 Corresponds to Src Expression

The sensitivity of primary GBM cell cultures to Si306, pro-Si306, and the well-known SFK inhibitor dasatinib after 72 h treatment was assessed by the MTT assay and compared to the results we previously obtained in U87 and U87-TxR cell lines (Table 1) [28]. When compared to commercial cell lines, the primary cultures were more resistant to all tested STKIs (Table 1). Importantly, cell lines as well as GBM-5 and GBM-6 were more sensitive to Si306 and pro-Si306 than to dasatinib. Only GBM-4 showed similar sensitivity to all tested STKIs.

**Figure 1:**
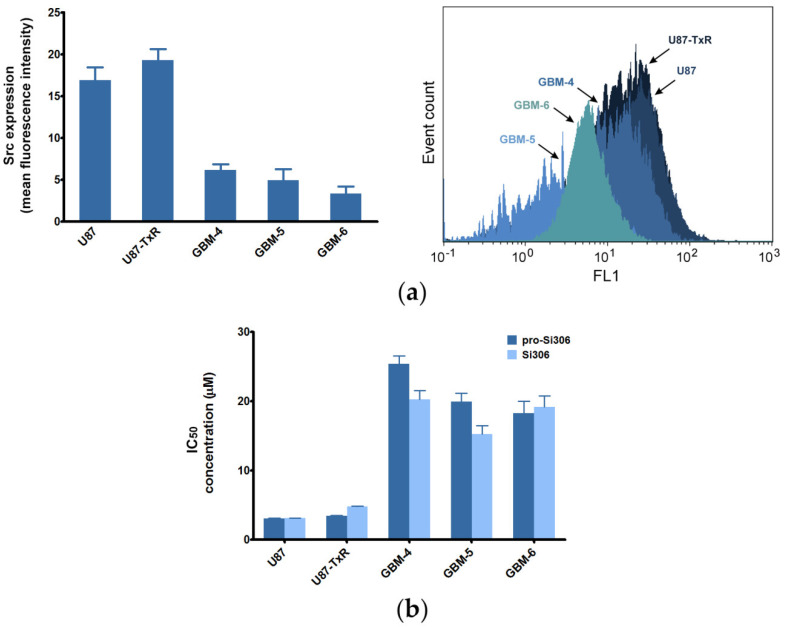
Sensitivity of GBM cells to Si306 and pro-Si306 and its relation to Src protein expression. (**a**) Histogram and flow-cytometric profiles representing Src expression in GBM cell lines (U87 and U87-TxR) and primary GBM cells (GBM-4, GBM-5 and GBM-6). Histogram shows mean fluorescence intensity (arbitrary units) obtained by flow-cytometric detection of immunolabeled cells. Values are expressed as mean ± SEM. (**b**) Sensitivity of GBM cell lines (U87 and U87-TxR) [28] and primary GBM cells (GBM-4, GBM-5 and GBM-6) to Src inhibitors Si306 and pro-Si306 expressed as half maximal inhibitory concentration (IC_50_) values ± SD (*n* = 3). The IC_50_ values were determined using the 3-(4,5-Dimethyl-2-thiazolyl)-2,5-diphenyl-2H-tetrazolium bromide (MTT) assay.

To assess the difference in the expression of Src in GBM cell lines and primary GBM cultures, we performed flow cytometry. The results showed that Src expression levels (Figure 1a) can be related to the IC_50_ values and sensitivity of GBM cells to the investigated agents (Figure 1b) [28]. Namely, higher sensitivity of GBM cells to Src inhibitors corresponded to higher expression of the target molecule.

### 2.2. Si306 and Pro-Si306 Decrease Migration and Invasion in GBM Cells

The migratory potential of U87 and U87-TxR cells after treatment with Si306 and pro-Si306 was assessed by wound healing assay. The 5 µM concentration of STKIs did not affect cellular metabolic activity after 24 h as determined by the MTT assay and was therefore selected for migration and invasion studies (Figure S1). Upon the application of Si306, the migration was significantly decreased in both cell lines (Figure S2). Likewise, although not statistically significant, the prodrug treatment displayed an anti-migratory trend.

**Figure 2:**
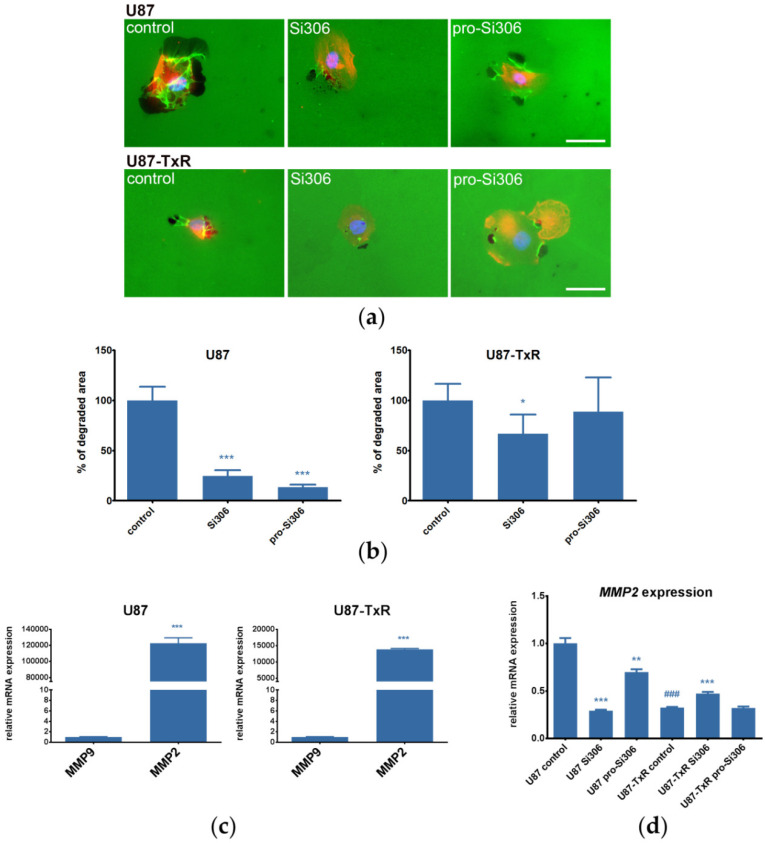
Si306 and pro-Si306 decrease the ability of GBM cell lines to degrade the extracellular matrix (ECM). (**a**) Representative images of gelatin degradation by U87 and U87-TxR cells treated with 5 µM Si306 and pro-Si306 for 24 h. Scale bar = 30 µm. (**b**) Percentage of area degraded by U87 and U87-TxR cells. (**c**) Relative expression of matrix metalloproteinases *MMP2* and *MMP9* in U87 and U87-TxR cells. (**d**) Relative expression of *MMP2* in U87 and U87-TxR cells treated with 5 µM Si306 and pro-Si306 for 24 h. All values are expressed as mean ± SEM (*n* = 3). Statistical significance between treated and control group is shown as * (*p* < 0.05), ** (*p* < 0.01), and *** (*p* < 0.001). Statistical significance between untreated cell lines is shown as ### (*p* < 0.001).

Next, the gelatin degradation assay was carried out to study the ability of U87 and U87-TxR cells to degrade the ECM upon treatment with 5 µM Si306 and pro-Si306. The STKIs showed a similar trend in decreasing the potential of U87 cells to degrade the ECM. In this cell line, the degradation of gelatin was decreased approximately 80% by both compounds, whereas in U87-TxR cells, the compounds were less effective (Figure 2a,b). A higher concentration of STKIs (10 µM) was also tested in U87 and U87-TxR cells, however no significant dose-response effects on gelatin degradation were observed, apart from U87-TxR cells treated with 10 µM pro-Si306 (Figure S3). 

Moreover, we assessed the mRNA expression of matrix metalloproteinases MMP-2 and MMP-9, enzymes responsible for the gelatin degradation (Figure 2c). The *MMP9* expression was very low in both cell lines suggesting that their gelatin degradation ability is more dependent on MMP-2 activity. Additionally, we observed that *MMP2* mRNA expression in U87 cells was notably higher when compared to U87-TxR cells (Figure 2c) which is line with their 10-fold higher ability to degrade gelatin (Figure S4a). The treatment with Si306 and pro-Si306 significantly decreased the *MMP2* mRNA expression in U87 cell line, supporting the gelatin degradation findings (Figure 2d).

**Figure 3:**
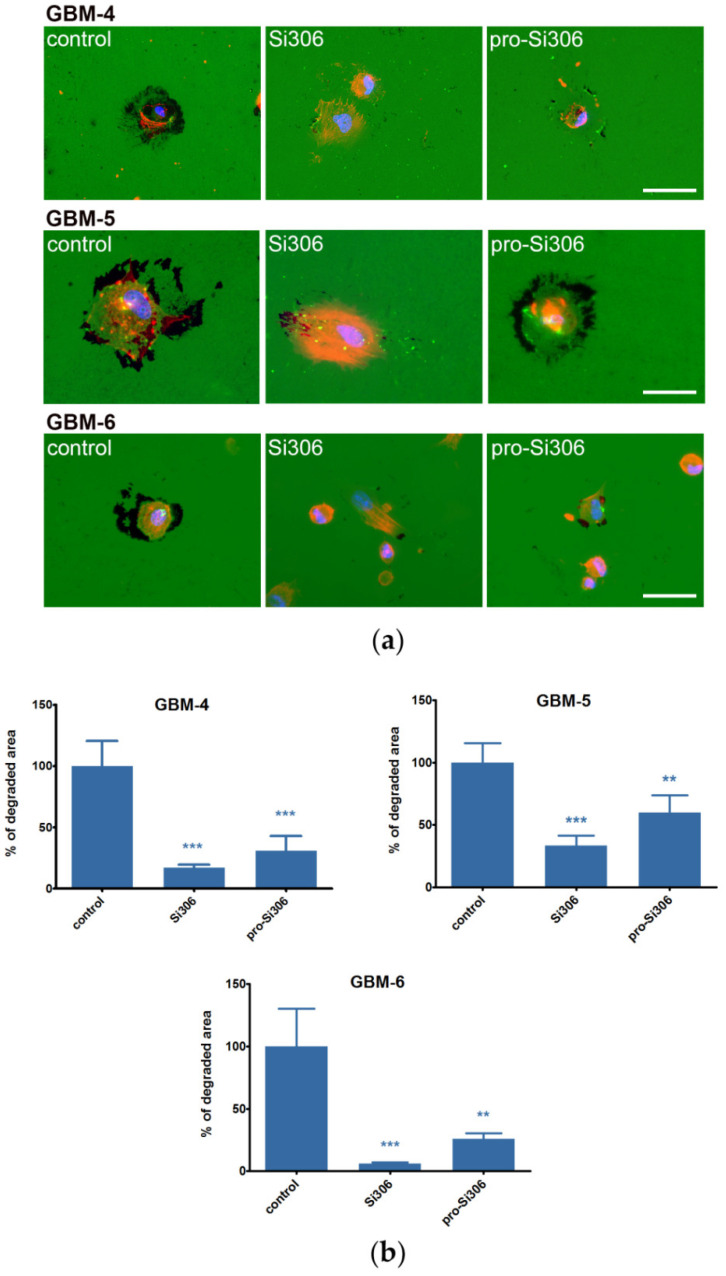
Si306 and pro-Si306 decrease the ability of primary GBM cells to degrade the ECM. (**a**) Representative images of gelatin degradation by primary GBM-4, GBM-5, and GBM-6 cells treated with 10 µM Si306 and pro-Si306 for 24 h. Scale bar = 30 µm. (**b**) Percentage of area degraded by primary GBM-4, GBM-5, and GBM-6 cells. Values are expressed as mean ± SEM (*n* = 3). Statistical significance between treated and control group is shown as ** (*p* < 0.01) and *** (*p* < 0.001).

The ability of primary GBM cultures to degrade the ECM was also studied by the gelatin degradation assay. To maintain the experimental conditions of the assay uniform for all GBM cells, primary cells were cultured and treated in 10% fetal bovine serum (FBS)-containing media, equivalent to the cell lines. When compared to U87 and U87-TxR cell lines, primary GBM cells showed greater potential to degrade the ECM (Figure S4a). GBM-4 and GBM-5 degraded gelatin more extensively than both cell lines, while GBM-6 potency was significantly lower. Upon treatment with non-cytotoxic concentrations of STKIs (below their IC_50_ values), gelatin degradation in GBM-4 cells decreased over 70% (Figure 3). In GBM-5 cells, Si306 treatment reduced gelatin degradation over 60%, while pro-Si306 also caused a notable decrease. In GBM-6, both STKIs, particularly Si306, nearly entirely blocked the degradation of gelatin (Figure 3). A higher concentration of STKIs (20 µM) was also tested in all primary GBM cultures, and apart from GBM-5 cells, we did not observe a significant dose-response effect on gelatin degradation (Figure S3).

**Figure 4:**
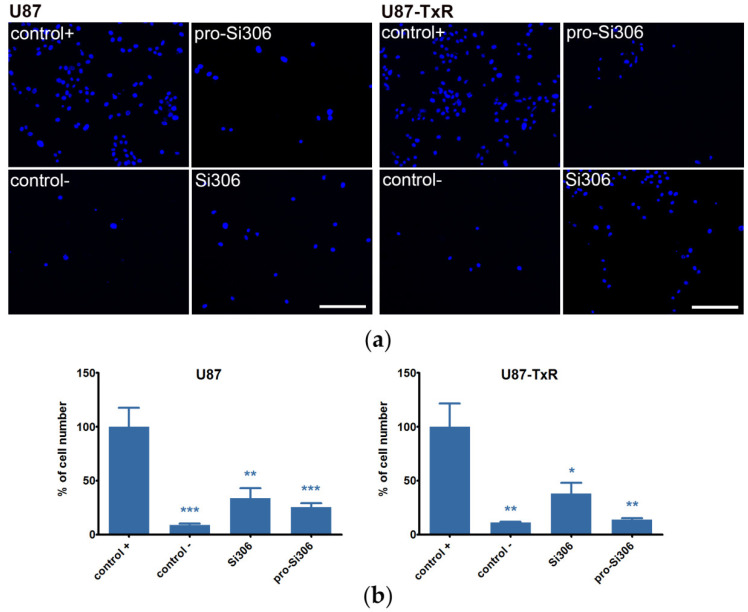
Si306 and pro-Si306 suppress GBM cell lines invasion through the basement membrane. (**a**) Representative images of U87 and U87-TxR cells that invaded through matrigel to the opposite side of the membrane after 24 h treatment with 5 µM Si306 and pro-Si306. Scale bar = 200 µm. (**b**) Percentage of U87 and U87-TxR cells that invaded through matrigel and passed the membrane. Values are expressed as mean ± SEM (*n* = 3). C+ is positive control (with fetal bovine serum (FBS) as chemo-attractant) and C- is negative control (without chemo-attractant). Statistical significance compared to positive control group is shown as * (*p* < 0.05), ** (*p* < 0.01), and *** (*p* < 0.001).

Furthermore, the investigated STKIs decreased the potential of U87 and U87-TxR cell lines to invade through the basement membrane in the matrigel invasion assay (Figure 4). The invasiveness comparison between GBM cell lines revealed that U87 has greater potential to intravade or extravade compared to U87-TxR (Figure S4b). In addition, we found U87 cells to contain more active phosphorylated forms of Src pathway components, which are known to be involved in invasion (Figure S4c). Regardless of the differences in U87 and U87-TxR invasive potential, treatment with both STKIs decreased their respective invasiveness over 50% (Figure 4).

### 2.3. Si306 and Pro-Si306 Inhibit the Activity of Src and its Upstream Signaling Pathway Components FAK and EGFR

**Figure 5:**
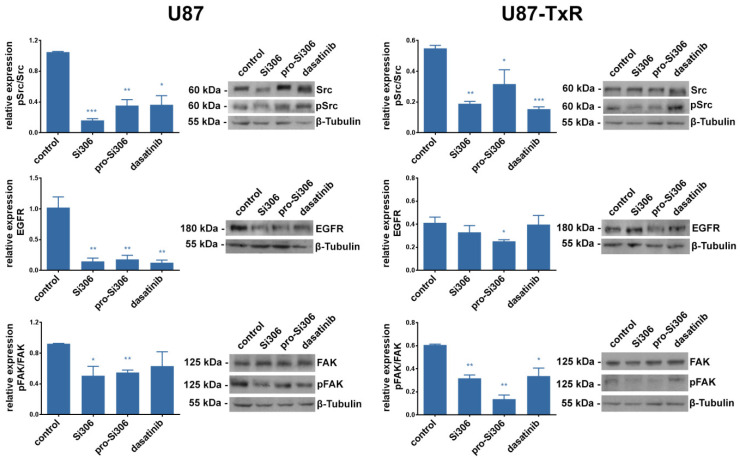
Si306 and pro-Si306 inhibit the activity of Src and its upstream signaling pathway components in GBM cell lines. Representative Western blot images of pSrc, Src, epidermal growth factor receptor (EGFR), pFAK, and focal adhesion kinase (FAK) protein expression in U87 and U87-TxR cells after 24 h treatment with 5 µM Si306 and pro-Si306 are shown. Histograms represent Western blot data expressed as phosphoprotein level relative to total protein level (Src and FAK) or total protein expression (EGFR), all normalized to β-tubulin. Values are expressed as mean ± SEM (*n* = 3). Statistical significance between treated and control group is shown as * (*p* < 0.05), ** (*p* < 0.01), and *** (*p* < 0.001), uncropped western blot in Figure S8.

To investigate whether the observed anti-invasive effects of Si306 and pro-Si306 are associated with Src signaling, its pathway components were evaluated by Western blot in GBM cell lines. We assessed the levels of Src and its active phosphorylated form pSrc, as well as the upstream members of the pathway: EGFR, FAK and its active phosphorylated form pFAK (Figure 5). The activity of Src, shown as pSrc/Src relative expression, was significantly decreased by STKIs in both GBM cell lines. In U87 cells, Src activity was reduced by 85% and 65% after Si306 and pro-Si306 treatment, respectively. Although less pronounced, the pSrc/Src relative expression in U87-TxR was also significantly reduced.

After treatment with STKIs, the relative expression of EGFR was decreased by over 80% in U87 cells. EGFR expression was significantly reduced in U87-TxR only after pro-Si306 exposure. The pFAK/FAK relative expression in U87 cells was decreased over 40% upon treatment with Si306 and its prodrug (Figure 5). In U87-TxR cells, Si306 decreased the activity of FAK by 50%, while its prodrug was even more effective. Importantly, Si306 and pro-Si306 showed equivalent or significantly stronger effects on Src and EGFR inhibition compared to dasatinib. However, the effect of Si306 and pro-Si306 on FAK inhibition was more pronounced.

### 2.4. Si306 and Pro-Si306 Inhibit the Activity of Src Downstream Signaling Pathway Components AKT and ERK

**Figure 6:**
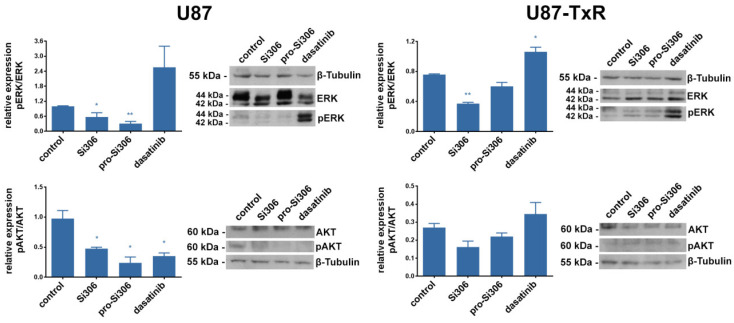
Si306 and pro-Si306 inhibit the activity of Src downstream signaling pathway components in GBM cell lines. Representative Western blot images of pERK, extracellular signal-regulated kinase (ERK), pAKT and protein kinase B (AKT) protein expression in U87 and U87-TxR cells after 24 h treatment with 5 µM Si306 and pro-Si306 are shown. Histograms represent Western blot data expressed as phosphoprotein level relative to total protein level, normalized to β-tubulin. Values are expressed as mean ± SEM (*n* = 3). Statistical significance between treated and control group is shown as * (*p* < 0.05), ** (*p* < 0.01), and *** (*p* < 0.001), uncropped western blot in Figure S8.

We also assessed the expression levels of the Src downstream pathway members: ERK, AKT and their active forms pERK, and pAKT (Figure 6). ERK activity was decreased in U87 cells by 40% and 70% after treatment with Si306 and its prodrug, respectively. In U87-TxR, the effect of both compounds on pERK/ERK relative expression was less pronounced. The activity of AKT in U87 cells was decreased by 50% and 75% upon Si306 and pro-Si306 application, respectively (Figure 6). The effect of Si306 was similar in U87-TxR cells, while pro-Si306 showed a trend of decreasing the pAKT/AKT relative expression. Both compounds showed significantly stronger effect on Src downstream signaling components compared to dasatinib. Dasatinib was not able to cause the ERK inhibition and only affected the activity of AKT in U87 cells. Moreover, dasatinib stimulated the activity of ERK in both cell lines.

### 2.5. Si306 and Pro-Si306 Inhibit Src Activity in Primary GBM Cells

**Figure 7:**
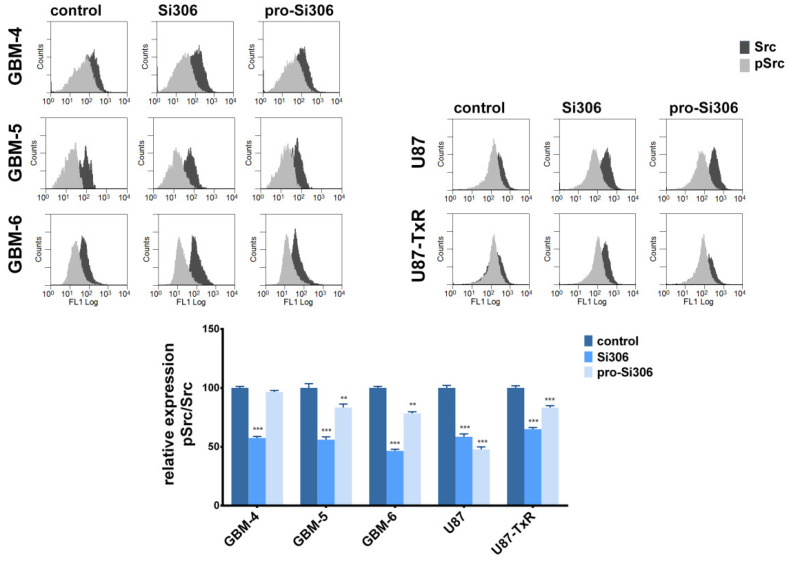
Si306 and pro-Si306 inhibit Src activity in primary GBM cells. Flow-cytometric profiles of Src and pSrc expression in primary GBM, U87, and U87-TxR cells treated with Si306 and pro-Si306. GBM-4, GBM-5, and GBM-6 cells were treated with 10 µM Si306 and pro-Si306 for 24 h, while U87 and U87-TxR cells were treated with 5 µM Si306 and pro-Si306 for 24 h. Histogram represents relative pSrc/Src expression. Values are expressed as mean ± SEM. Statistical significance between treated and control group is shown as ** (*p* < 0.01), and *** (*p* < 0.001).

To investigate whether the observed anti-invasive effects of Si306 and pro-Si306 are associated with Src activity in primary GBM cell cultures, the levels of Src and pSrc were determined by flow cytometry. Primary cells were treated for 24 h with 10 µM STKIs, as this treatment prominently reduced gelatin degradation. The pSrc/Src relative expression was decreased by 40% after Si306 treatment in GBM-4 and GBM-5 cells (Figure 7). Si306 also decreased Src activity in GBM-6 over 50% (Figure 7). Although not as efficient as Si306, pro-Si306 significantly decreased the pSrc/Src relative expression in GBM-5 and GBM-6. Next, the ability of Si306 and its prodrug to inhibit Src activity was compared between primary and commercial GBM cells (Figure 7). U87 and U87-TxR cells were treated with 5 µM STKIs for 24 h, as this treatment notably reduced their potential to degrade the ECM. Although the effect of STKIs on ECM degradation reduction was more prominent in primary GBM cells, the STKI’s potency to inhibit Src activity was largely comparable between primary and commercial GBM cells.

### 2.6. Si306 and Pro-Si306 Suppress Invasion of GBM Cells in Vivo

**Figure 8:**
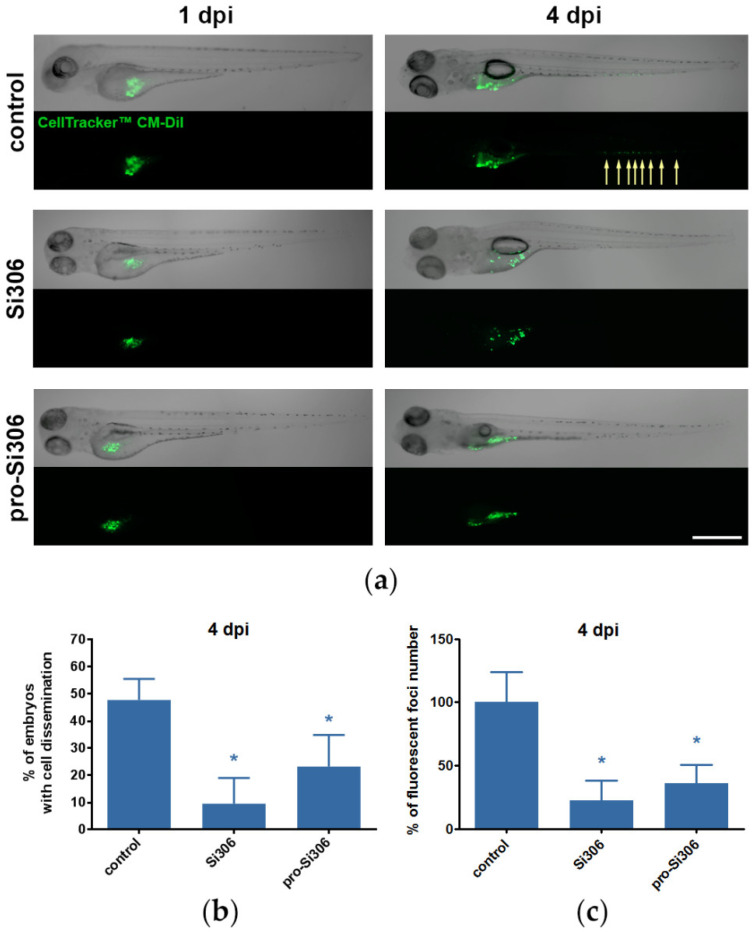
Si306 and pro-Si306 suppress invasion of GBM cells in vivo. (**a**) Representative panorama images of U87 xenografts in zebrafish embryo model at 1 dpi (before treatment) and 4 dpi (after 72 h treatment with 2.5 µM Si306 and pro-Si306). Arrows indicate dissemination of cells outside of the yolk sac. Scale bar = 500 µm. (**b**) Percentage of embryos with cell dissemination after 72 h treatment with Si306 and pro-Si306. (**c**) Percentage of fluorescent foci number in embryos displaying invasion after 72 h treatment with Si306 and pro-Si306. All values are expressed as mean ± SEM. Statistical significance between treated and control group is shown as * (*p* < 0.05).

Next, we evaluated the anti-invasive effects of Si306 and pro-Si306 in vivo in a zebrafish embryo GBM xenograft model. As the U87 cell line showed strong invasive and migratory potential in vitro, U87 xenografts were used for further in vivo experiments. U87 cells were microinjected into the yolk sac of 48 h post fertilization (hpf) embryos and 2.5 µM Si306 or pro-Si306 treatment was applied one day post injection (dpi). The 72-h treatment with STKIs did not induce cells loss in xenografts compared to control, as overall CM-Dil dye fluorescence remained constant between groups (Figure S5a). Dissemination of U87 cells from the injection site was monitored after 72 h at 4 dpi (Figure 8a). Xenografted cells exhibited invasive behavior and their capability of invading into the avascular caudal region was notably reduced upon STKI treatment (Figure S5b). After treatment with Si306, the percentage of embryos displaying invasion decreased by 80% compared to untreated control (Figure 8b). The same trend was also observed after pro-Si306 treatment, where the percentage of embryos with cell dissemination was reduced by 50%. Likewise, the number of disseminated fluorescent foci in 4 dpi embryos was significantly reduced after treatment with STKIs (Figure 8c).

## 3. Discussion

In this study, we evaluated the anti-invasive properties of pyrazolo[3,4-d]pyrimidine derivatives, Si306 and pro-Si306, as ATP-competitive Src tyrosine kinase inhibitors in GBM cells in vitro and in vivo. Our results show that both compounds, the drug and its prodrug, significantly suppressed GBM invasion by inhibiting the Src signaling pathway. Particularly, the inhibition of FAK, a kinase upstream to Src, coincided with reduced ECM degradation and reduced motility of GBM cells. 

Glioblastoma is the most common and the most aggressive primary brain tumor with a dismal prognosis, accounting for 45.6% of central nervous system (CNS) tumors [43,44]. One of the main challenges in GBM treatment is its resistance to current treatment protocols. Chemoresistance is mainly a consequence of high expression of ABC transporters in the BBB, which protect the brain from chemical damage [45]. Another reason for the intrinsic resistance is the ability of GBM to disseminate and invade through normal brain tissue [46].

Considering that we have not seen progress in establishing new therapeutic protocols for GBM, there is an urgent need for new agents against GBM that can cross the BBB and target invading GBM cells [7]. In recent years, Src inhibition has been in focus of different studies related to GBM treatment [14,47,48,49]. This approach seems to be rational due to the impact of Src on invasion, migration and proliferation of GBM [14]. Namely, Src activation mediates increased motility of GBM cells, altered adhesion and ECM remodeling [7]. Our results showed that primary GBM cells are less sensitive to STKIs compared to GBM cell lines U87 and U87-TxR, which may reflect the differences in the expression levels of the target molecule. Indeed, we observed that the abundance of Src tyrosine kinase is reciprocal to the efficacy of STKIs.

An invasive GBM phenotype is characterized by high ability of cancer cells to degrade and migrate through the ECM, which is associated with the activity of matrix metalloproteinases MMP-2 and MMP-9, responsible for the ECM remodeling [50,51,52]. MMP-2 and MMP-9 are primarily associated with the invasiveness of high-grade gliomas [53], and MMP-2 activity in GBM was reported to be five-fold greater compared to normal brain or low-grade gliomas [54].

We observed the inhibitory effect of both STKIs on gelatin degradation in GBM cell lines U87 and U87-TxR. However, the effect was more prominent in U87 cells, probably due to the higher expression of *MMP2* in this cell line. Moreover, both STKIs were able to significantly inhibit the *MMP2* expression in U87 which implies that Si306 and pro-Si306 may directly or indirectly target *MMP2*. 

Two out of three primary GBM cultures showed a remarkable ability to degrade the ECM, which is likely associated with high invasiveness. Nevertheless, STKIs showed stronger inhibitory effect on gelatin degradation in all established primary GBM cell cultures compared to commercial cell lines. Furthermore, in contrast to commercial cell lines, this prominent effect was achieved with inhibitor concentrations notably lower than IC_50_ values, which could imply that Src has a more important role in invasion than cell survival and growth in primary GBM.

The migration of GBM cells through the basement membrane includes intravasation and extravasation, processes regulated by various mechanisms different to those mediating ECM degradation [55]. Although U87 cells showed greater migration through the basement membrane compared to U87-TxR cells, Si306 and pro-Si306 successfully decreased invasiveness in both GBM cell lines showing the potential to suppress actin-related cellular motility.

To assess the mechanisms behind these findings, we investigated whether treatment with Si306 and pro-Si306 can reduce the levels of active Src in GBM cells, as well as upstream and downstream members of the Src signaling pathway. We assumed that the anti-invasive properties of studied STKIs are the result of the Src signaling components’ decreased activities, particularly the inhibition of FAK, as this kinase is one of the key regulators of cell migration and invasion. Earlier findings showed that FAK is responsible for coordination of cell motility and ECM remodeling during cancer cell invasion, and contributes to the secretion of matrix metalloproteinases [20]. The pharmacological inhibition of FAK phosphorylation was linked to reduced MMP-2 and MMP-9 expression and activation in breast and lung cancer cells [56]. Moreover, FAK was found to be crucial in promoting invasion and matrix metalloproteinases production in GBM [57,58]. In U87 and CCF-STTG1 GBM cells, the downregulation of FAK was associated with suppressed invasion via reduced MMP-2 and MMP-9 secretion [59]. Another study found that the inhibition of FAK phosphorylation in U373MG and A-172 GBM cells was accompanied by decreased migration and invasion and suppressed MMP-2 gelatinolytic activity and expression [60]. Therefore, the decrease in gelatinases’ activities that we observed in U87 cells after treatment with STKIs is likely associated with the reduction in FAK activity. 

Src has been shown to interact with EGFR, and has frequently been linked with the activation of this clinical GBM marker [61]. EGFR may be activated via transactivation, a mechanism where the presence of a ligand is not required [62]. In the absence of EGFR ligand, Src can mediate its transactivation by G protein-coupled receptors and other extracellular stimuli [61]. In addition, EGFR overexpression and decreased degradation were correlated with Src activity in cancer cells, leading to increased EGFR signaling in the presence of activated Src [63,64,65]. The observed reduction in EGFR protein levels after treatment with Si306 and pro-Si306 could be associated with diminished EGFR degradation as a result of reduced Src activity.

The efficacy of Si306 and pro-Si306 regarding Src signaling inhibition was compared to dasatinib, a well-studied SFK inhibitor. Dasatinib is known to exhibit anti-invasive effects in cancer cells by downregulating FAK phosphorylation [66,67,68]. The inhibitory effect of dasatinib on the ERK activity was also demonstrated in various cancer types [66,69,70,71]. Importantly, Si306 and pro-Si306 showed greater potency for FAK inhibition than dasatinib in our GBM cells, while dasatinib was not able to suppress the ERK activity. Dasatinib, however, did not achieve clinical success due to its poor pharmacokinetics [24,25,72]. On the contrary, Si306 and its prodrug have displayed encouraging pharmacokinetic features and tolerability in vivo [28] making them worthy of further development.

We previously showed that Si306 notably reduced the growth of U87 xenografts in nude mice, particularly in combination with radiotherapy [14], but the effects of Si306 and its prodrug on GBM invasion in vivo have not been studied up to now. Compared to mouse models, the xenotransplantation of cancer cells into a transparent zebrafish embryo enables the real-time monitoring of cell invasion and a significantly shorter observation period of this process [73,74]. In agreement with the results we obtained in vitro, Si306 and pro-Si306 displayed a strong anti-invasive effect and reduced GBM cell dissemination in vivo. Both STKIs decreased the percentage of zebrafish embryos displaying invasion, as well as the number of GBM cell dissemination foci.

## 4. Materials and Methods 

### 4.1. Drugs

Derivatives of the pyrazolo[3,4-d]pyrimidine, Si306 and its prodrug pro-Si306, were obtained as previously described [40]. Dasatinib was purchased from Sigma-Aldrich Chemie GmbH, Germany. Si306, pro-Si306, and dasatinib were dissolved in dimethyl sulfoxide (DMSO) and kept at room temperature as 20 mM aliquots. Immediately before treatments, drugs were diluted in sterile phosphate buffered saline (PBS).

### 4.2. Cell Lines

The human glioblastoma cell line U87 was purchased from American Type Culture Collection (Rockville, MD, USA). MDR U87-TxR cells with P-gp overexpression were previously selected in our laboratory from parental U87 cells after continuous exposure to increasing concentrations of paclitaxel [75]. Both cell lines were cultured in Minimum Essential Medium (MEM) (Biowest, France) supplemented with 10% FBS, L-glutamine (2 mM) and 10000 U/mL penicillin, 10 mg/mL streptomycin solution. Cells were maintained at 37 °C in humidified 5% CO_2_ atmosphere.

### 4.3. Primary Glioblastoma Cultures

Samples from patients with WHO grade IV glioblastoma were collected between April 2018 and May 2019 from the Clinic for Neurosurgery at the Clinical Center of Serbia. The histological grade was established by histopathological analysis of the surgical specimens. The samples were collected and used in the study after obtaining patients’ informed consents and the approval from the Ethics Committee of the Clinical Center of Serbia (ref. number 586/4), in accordance with the ethical standards laid down in the 1964 Declaration of Helsinki. Tissue samples were collected during surgery and immediately processed. 

First, the tissue was chopped with a surgical blade in a Petri dish in sterile conditions. Accumax solution (Sigma-Aldrich Chemie GmbH, Germany) was then added to the chopped tissue for 15 min at room temperature for chemical dissociation. Dissociated tissue was then centrifuged and resuspended in 5 mL of DMEM/F12 medium (Biowest, France), supplemented with 10% FBS, 2 mM L-glutamine, 10,000 U/mL penicillin, 10 mg/mL streptomycin, and 25 µg/mL amphotericin B solution. DMEM/F12 medium was additionally supplemented with growth factors: 20 µL/mL B-27 (Thermo Fisher Scientific, MA, USA), 40 ng/mL epidermal growth factor (Thermo Fisher Scientific, MA, USA), and 20 ng/mL basic fibroblast growth factor (Thermo Fisher Scientific, MA, USA). Dissociated tissue was monitored for 48 h to confirm cell attachment before changing the medium. Attached cells were grown until confluence prior to further investigation. Correspondingly to cell lines, primary cells were maintained at 37 °C in humidified 5% CO_2_ atmosphere. We kept low passage number to prevent genetic and epigenetic changes in cells [76], which is crucial to ensure the translational potential of obtained results in primary cultures [77].

### 4.4. Immunocytochemistry of Primary Glioblastoma Cultures

After establishing the primary cell culture, 25,000 cells per chamber were seeded in 4-well chamber slides (Nunc, Nalgene, Denmark) in 500 µL of medium. After 24 h, cells were washed in PBS, fixed in 4% paraformaldehyde (PFA) for 15 min and blocked in 0.5% bovine serum albumin (BSA) in PBS for 1 h at room temperature. 

To verify their GBM origin, the cells were incubated with 1:500 dilutions of primary monoclonal mouse anti-vimentin and polyclonal rabbit anti-glial fibrillary acidic protein (GFAP) antibody (DAKO, CA, USA) overnight at 4 °C. After washing in PBS, secondary antibodies Alexa Fluor 488 anti-mouse IgG (H + L) (Cell Signaling Technology, MA, USA) and Alexa Fluor 555 anti-rabbit IgG (H + L) (Cell Signaling Technology, MA, USA) were applied at 1:1000 dilutions in 0.5% BSA in PBS for 1 h at room temperature. Cells were also co-stained with Hoechst 33342 to mark the nuclei and mounted in Mowiol. Subsequently, cells were visualized at 20× magnification under a Zeiss Axiovert inverted fluorescent microscope (Carl Zeiss Foundation, Germany) equipped with AxioVision 4.8 Software. Three primary cell cultures GBM-4, GBM-5, and GBM-6 expressed both vimentin and GFAP (Figure S6). Since co-expressed vimentin and GFAP are markers of GBM tumors, the established primary cultures were considered as GBM cell cultures.

### 4.5. Flow Cytometry

Src and pSrc protein levels in U87 and U87-TxR cell lines and primary GBM cells were assessed via flow cytometric analysis. Cells were seeded into 6-well cell culture plates (200,000 cells per well) and incubated overnight in 1 mL of appropriate medium. Then, U87 and U87-TxR cells were treated with 5 µM Si306 or pro-Si306, while primary GBM cells were treated with 10 µM STKIs. 

Cells were harvested after 24 h, washed in PBS, and fixed in 4% PFA for 10 min at room temperature. Cells were then permeabilized by adding ice cold 90% methanol for 30 min at 4 °C. After washing in PBS, cells were incubated with 0.5% BSA in PBS for 1 h at room temperature for blocking of unspecific binding. Cells were then incubated overnight at 4 °C with primary rabbit monoclonal anti-Src or anti-pSrc antibodies (Cell Signaling Technology, MA, USA) diluted 1:100 in 0.5% BSA in PBS. After washing in PBS, cells were incubated with secondary antibody Alexa Fluor 488 anti-rabbit IgG(H + L) (Sigma-Aldrich Chemie GmbH, Germany) diluted 1:1000 in 0.5% BSA in PBS and incubated for 30 min at room temperature. Cells were subsequently washed and resuspended in 1 mL of PBS. Unlabeled cells served as background fluorescence control, and cells labeled with rabbit monoclonal antibody IgG XP® isotype control (Cell Signaling Technology, MA, USA) served as unspecific binding control (Figure S7). The fluorescence intensity was measured in FL1-H channel on flow cytometer (Partec, Münster, Germany) and data were analyzed by Summit 4.3 (DAKO, CA, USA) and FCSalyzer 0.9.17 software (https://sourceforge.net/projects/fcsalyzer/).

### 4.6. Western Blot Analysis

The protein levels in U87 and U87-TxR cells were assessed via Western blot analysis. Cells were seeded into cell culture flasks (1 × 10^6^ cells per flask) and incubated overnight in 5 mL of MEM medium. Cells were then treated with 5 µM Si306 or pro-Si306 for 24 h. Next, 1x10^6^ cells per sample were lysed in Laemmli buffer (glycerol, 1M TRIS pH 6.8, 1% SDS, mQH_2_O and 20% β-mercaptoethanol) with bromphenol blue. Samples were then boiled for 5 min at 95 °C and kept at -80 °C until usage. The normalization (standardization) of protein amounts loaded onto the gel for Western blotting was completed by separation of equal volumes of protein samples on SDS-PAGE and followed by Coomassie Brilliant Blue staining. The equal amounts of proteins were then run on 8% or 12% SDS-PAGE and proteins were subsequently transferred to PVDF membrane (Immobilon®-PSQ, Merck Millipore, Ireland). The membranes were blocked with 5% BSA Tris-buffered saline/0.1% Tween-20 (TBST) for 1 h at room temperature and incubated overnight at 4 °C with the following primary rabbit antibodies: polyclonal anti-EGFR (Thermo Fisher Scientific, MA, USA; 1:750), monoclonal anti-FAK (Cell Signaling Technology, MA, USA; 1:1000), monoclonal anti-pFAK (Thermo Fisher Scientific, MA, USA; 1:1000), monoclonal anti-Src (Cell Signaling Technology, MA, USA; 1:5000), monoclonal anti-pSrc (Cell Signaling Technology, MA, USA; 1:1000), monoclonal anti-ERK (Thermo Fisher Scientific, MA, USA; 1:5000), monoclonal anti-pERK (Thermo Fisher Scientific, MA, USA; 1:500), monoclonal anti-AKT (Cell Signaling Technology, MA, USA; 1:1000), monoclonal anti-pAKT (Cell Signaling Technology, MA, USA; 1:750). After incubation with primary antibodies, membranes were rinsed for 5 min, 6 times in TBST and incubated for 1 h at room temperature with a Horse Radish Peroxidase (HRP)-conjugated bovine anti-rabbit secondary antibody (Abcam, UK, 1:5000 and1:10,000). Immunoreactivity was detected by enhanced chemiluminiscence (ECL, GE Healthcare) and exposed on X-ray film. Each blot has been re-probed with rabbit monoclonal anti-β-tubulin (Sigma-Aldrich Chemie GmbH, Germany, 1:50,000) antibody and incubated with (HRP)-conjugated anti-rabbit secondary antibody (Abcam, UK, 1:10,000). All antibodies were diluted in TBST. Signals were quantified by densitometry using ImageJ software (U.S. National Institutes of Health, Bethesda, MD, USA) and expressed as relative values (i.e., density ratio normalized to the corresponding internal control, β-tubulin signal).

### 4.7. MTT Assay

The U87, U87-TxR and primary GBM cells were seeded into flat-bottomed 96-well cell culture plates (4000 cells per well) and incubated overnight in 100 µL of the appropriate medium. U87 and U87-TxR cells were treated for 24 h with increasing concentrations of Si306 and pro-Si306 (2.5, 5, and 10 µM) to assess cellular metabolic activity. Primary GBM cells were treated for 72 h with increasing concentrations of Si306, pro-Si306 and dasatinib (1, 2.5, 5, 10, and 25 µM). This colorimetric assay is based on the 3-(4,5-Dimethyl-2-thiazolyl)-2,5-diphenyl-2H-tetrazolium bromide (MTT) enzymatic reduction into formazan dye by active mitochondria in viable cells, indicative of their metabolic activity. The insoluble formazan crystals are dissolved with a solubilization solution and the resulting purple-colored solution is quantified by measuring absorbance. MTT was purchased from Sigma-Aldrich Chemie GmbH, Germany. After treatment, 100 μL of MTT solution (2 mg/mL) was added to each well and the plates were incubated at 37 °C for 4 h. Subsequently, formazan product formed in cells with intact/viable mitochondria was dissolved in 200 µL of DMSO and absorbance was measured at 540 nm using an automated microplate reader (LKB 5060–006 Micro Plate Reader, LKB, Vienna, Austria). The IC_50_ values for Si306, pro-Si306, and dasatinib were calculated by non-linear regression analysis using GraphPad Prism 6.0 software (GraphPad Software, La Jolla, CA, USA).

### 4.8. Wound Healing Assay

The migratory potential of U87 and U87-TxR cell lines was evaluated by the wound healing assay. Cells were seeded in 24-well cell culture plates (50,000 cells per well) and grown overnight. After reaching confluence, a uniform wound was scratched into a monolayer of each well with a sterile 200 μL micropipette tip. Next, medium was replaced and cells were treated with 5 µM Si306 or pro-Si306. Immediately after treatment, cells were imaged by Nikon Eclipse TS100 microscope equipped with a Nikon Coolpix 5000 camera (Nikon Instruments Inc., Amstelveen, Netherlands). Wound closure was monitored 24 h after wounding. First, cells were washed in PBS and fixed in 4% PFA for 30 min. Cells were then incubated with 1% crystal violet in 2% ethanol for 30 min, subsequently washed in PBS and imaged. The captured images were analyzed by ImageJ software to measure the degree of closure of the wounded area.

### 4.9. Gelatin Degradation Assay

The potential of GBM cells to degrade the ECM, which corresponds to their migratory ability, was assessed by gelatin degradation assay. U87, U87-TxR and primary GBM cells were seeded in 6-well cell culture plates (50,000 cells per well) on glass coverslips coated with fluorescently labeled gelatin (Gelatin from Pig Skin, Oregon Green® 488 Conjugate, Thermo Fisher Scientific, MA, USA). U87 and U87-TxR cells were treated with 5 µM and 10 µM Si306 and pro-Si306, while primary GBM cultures were treated with 10 µM and 20 µM Si306 and pro-Si306. After 24 h, cells were fixed with 4% PFA in PBS and co-stained with Hoechst 33342 (Sigma-Aldrich Chemie Gmbh, Germany) to mark the nuclei and ActinRed™ 555 ReadyProbes™ Reagent (Thermo Fisher Scientific, MA, USA) to mark actin filaments. Cells and degraded area of gelatin were visualized at 20× magnification under a Zeiss Axiovert inverted fluorescent microscope. Size of the dark area under cells that corresponds to gelatin degradation was measured in ImageJ software and normalized to the number of nuclei in each image. At least 100 cells were analyzed per experiment.

### 4.10. Transwell Invasion Assay

The effect of Si306 and pro-Si306 on the ability of U87 and U87-TxR cells to degrade the matrix and invade through basement membrane was evaluated by matrigel invasion assay using Transwell inserts (membrane pore size, 8 µm; diameter, 6.4 mm; BD Biosciences Discovery Labware, MA, USA). The cells were plated in a serum free medium in the upper chambers (150,000 cells/chamber) covered with a layer of 500 g/mL Matrigel (Corning Inc., NY, USA), a solubilized basement membrane preparation rich in ECM proteins laminin, collagen IV, heparin sulfate proteoglycans, entactin/nidogen and growth factors, and immediately treated with 5 µM Si306 and pro-Si306. The lower chambers were filled with MEM medium containing 10% FBS as a chemo-attractant. The control of spontaneous cell invasion in medium without 10% FBS was also included. After 24 h, cells that migrated through the membrane were fixed in 4% PFA in PBS, stained with Hoechst 33342 and counted under a Zeiss Axiovert inverted fluorescent microscope at 10× magnification. The average number of cells per membrane was analyzed by ImageJ software.

### 4.11. RNA Extraction and Reverse Transcription Reaction

The total RNA was isolated from U87 and U87-TxR cell lines. Cells were treated with 5 µM Si306 and pro-Si306 for 24 h. Isolation was performed with Trizol® reagent (Termo Fisher Scientific, MA, USA) according to the manufacturer’s instructions. Spectophotometry was used for RNA quantification. RNA quality was evaluated by agarose gel electrophoresis. Subsequently, reverse transcription reaction was carried out using 2 µg of total RNA, with a high-capacity cDNA reverse transcription kit (Applied Biosystems, Carlsabad, CA, USA) according the manufacturer’s instructions.

### 4.12. Quantitative Real-Time PCR

The expression levels of *MMP2* (forward primer 5’-TTC TTC GCA GGG AAT GAG-3’; reverse primer 5’-ACG ACA GCA TCC AGG TTA T-3’) and *MMP9* (forward primer 5’-AAA TGT GGG TGT ACA CAG GC-3’; reverse primer 5’-TTC ACC CGG TTG TGG AAA CT-3’) [78] were analyzed by quantitative real time PCR (qPCR). Reactions were performed by Maxima SYBR Green/ROX qPCR Master Mix (Thermo Scientific, MA, USA) in a QuantStudio 3 Real-Time PCR System (Thermo Fisher Scientific, MA, USA) according to the manufacturer’s recommendations, using 100 ng cDNA and primers specific for each gene and ACTB1 as internal control for normalization. Each sample was tested in triplicate and relative gene expression was analyzed by 2−ΔΔCt method [79].

### 4.13. Zebrafish Husbandry

The zebrafish embryo model is a valuable tool in drug screening and cancer research as it enables rapid and reproducible results, and is particularly suitable for studying invasiveness, metastasis and neoangiogenesis [80]. Zebrafish embryos are permeable for small molecules and therefore a good platform for evaluating candidate drugs.

For the GBM xenograft model, we used Tübingen wild-type zebrafish (*Danio rerio*) strain. Maintenance, embryo collection, staging and incubation were conducted according to standard procedures and guidelines (The Zebrafish Book) [81]. All animal handling was in accordance with local and national regulations with obtained Ethics Approval by the Ministry of Agriculture, Forestry and Water Management of the Republic of Serbia—Veterinary Directorate (ref. number 323-07-02116/2020-05/1).

Fish were maintained in fish medium (2 mM CaCl_2_, 0.5 mM MgSO_4_, 0.7 mM NaHCO_3_, 0.07 mM KCl) at a temperature of 27 ± 1 °C under continuous water aeration and filtering, and under artificial light with a 14:10 h dark/light cycle. Males and females were kept apart and regularly fed twice a day with commercial dry-flake food (TetraMin™ flakes; Tetra Melle, Germany) supplemented with *Artemia* nauplii. The day before spawning, males and females were placed in a breeding tank at a ratio of 1:2 before the onset of darkness and left undisturbed overnight. At the onset of light, the separators were removed from the breeding tanks. After 30 min, the eggs were collected, rinsed twice from debris using fresh embryo medium (Instant ocean®), and transferred into Petri dishes containing the embryo medium. Collected embryos were incubated in embryo medium until 48 hpf. During the first 24 hpf, 0.003% 1-phenyl-2-thiourea (PTU) (Sigma-Aldrich Chemie GmbH, Germany) was added to embryo medium in order to stop the development of pigmentation.

### 4.14. Zebrafish Xenograft Assay

The zebrafish embryos were staged for cell xenotransplantation at 48 hpf. Embryos were de-chorionized using micro-forceps, anesthetized in a 1:10 dilution of 0.003% Tricaine-S (Western Chemicals Inc, AZ, USA) and positioned on a wet 1.0% agarose pad for cells injection. U87 cells were harvested by trypsinization and washed with PBS at room temperature. Cells were labeled with 2.5 µl/mL CellTracker™ CM-DiI Dye (Thermo Fisher Scientific, MA, USA) diluted in PBS. Cells were then incubated in the dark for 4 min at 37 °C followed by 15 min at 4 °C and washed twice with PBS. Subsequently, cells were counted, resuspended in MEM medium and approximately 125 cells per embryo were injected into the center of the yolk sac with a microinjector (FemtoJet 4i, Eppendorf, Germany) equipped with borosilicate glass capillaries (Femtotips, Eppendorf, Germany) and micromanipulator (MM-3, Narishige, Japan). Next, the injected embryos were observed under a fluorescent microscope Olympus BX51 (Olympus Corporation, Japan) and only successfully injected embryos were used further for experiments. Selected embryos were transferred to a 96-well cell culture plate (one embryo per well) containing 200 µL of embryo medium containing PTU and maintained at 32 °C 

After 24h, at 1 dpi, the injected embryos were imaged with a fluorescent microscope and divided into three groups: control and groups treated with 2.5 µM Si306 or pro-Si306. Each group contained 32 embryos. After 72 h, at 4 dpi, each embryo was imaged again with a fluorescent microscope and the percentage of embryos exhibiting cancer cell dissemination from the injection site, the number of disseminated cells, and fluorescence intensity of CM-DiI-labeled xenografted cells were quantified in ImageJ software. Invasion was defined as 5 or more disseminated cells found outside of the vasculature [82,83]. The experiment was repeated three times.

### 4.15. Statistical Analysis

Statistical analyses were performed by GraphPad Prism 6.0 software. The normality of the data was estimated by Shapiro–Wilk’s test. The data obtained by wound healing assay were analyzed by one-way analysis of variance (ANOVA) test. Gelatin degradation assay data did not have a normal distribution, so the Mann–Whitney compare ranks test was carried out. The data obtained by matrigel invasion assay and flow cytometry were analyzed by unpaired Student’s t-test, while data obtained by Western blot, qPCR and zebrafish xenotransplantation assay were analyzed by paired Student’s t-test. The accepted level of significance was *p* < 0.05.

## 5. Conclusions

We demonstrated that the investigated Src inhibitors, Si306 and its prodrug pro-Si306, significantly decreased the invasive potential of human GBM cell lines and primary GBM cultures in vitro. The novel Src inhibitors were notably effective in patient-derived GBM cultures with a high ability for ECM degradation. The evaluation of STKI effects in different primary GBM cultures provides the potential for future personalized approaches in therapy. Compared to commercial cell lines, primary GBM cell cultures more accurately represent the heterogeneity of tumors in patients, and the findings we obtained after targeting Src in different primary GBM cultures could benefit the further optimization of investigated STKIs. 

Importantly, we found that both compounds suppress the activity of FAK, an important contributor to the invasive phenotype of GBM cells. Furthermore, Si306 and pro-Si306 successfully diminished GBM invasiveness in vivo in a zebrafish embryo xenograft model. Considering their characteristics as BBB penetrating agents with strong anti-invasive potentials, Si306 and pro-Si306 could be valuable candidates for GBM-targeted therapy. It is noteworthy that pro-Si306 showed similar efficacy to its corresponding drug in our experimental settings. This finding should stimulate further preclinical and clinical investigations of the prodrug due to its ability to provide longer availability and the exposure of the brain tissue to the drug.

## Figures and Tables

**Table 1:** Sensitivity of glioblastoma (GBM) cells to Src tyrosine kinase (Src) inhibitors after 72 h treatment expressed as IC_50_ values (µM).

Compounds	U87 ^1^	U87-TxR ^1^	GBM-4	GBM-5	GBM-6
Si306	3.081 ± 0.260	4.775 ± 0.322	20.270 ± 1.225	15.250 ± 1.207	19.120 ± 1.652
Pro-Si306	3.045 ± 0.343	3.419 ± 0.359	25.390 ± 1.159	19.920 ± 1.196	18.280 ± 1.696
Dasatinib	6.143 ± 0.464	8.516 ± 0691	18.60 ± 1.259	25.610 ± 1.216	25.350 ± 1.199

^1^ Data taken from [28].

## Supplementary Materials

The following are available online at https://www.mdpi.com/2072-6694/12/6/1570/s1, Figure S1: Sensitivity of U87 and U87-TxR cell lines to Si306 and pro-Si306 after 24 h treatment, Figure S2: Anti-migratory effect of Si306 and pro-Si306 in GBM cell lines, Figure S3: Si306 and pro-Si306 decrease the ability of GBM cells to degrade the ECM, Figure S4: Invasive potential of GBM cells, Figure S5: The effects of Si306 and pro-Si306 on GBM xenografts cell loss and invasion, Figure S6: Characterization of primary GBM cultures, Figure S7: Flow cytometry controls for immunolabeled GBM cells, Figure S8: Western blots from Figure 5 and Figure 6 representing in Scr, pScr, EGFR, FAK, pFAK, ERK, pERK, AKT and pAKT in U87 and U87-Txr cells with 5 uM Si306, pro-Si306, and dasatinib for 24 h.
