# Supplementary material for: Src Inhibitors Pyrazolo[3,4-d]pyrimidines, Si306 and Pro-Si306, Inhibit Focal Adhesion Kinase and Suppress Human Glioblastoma Invasion In Vitro and In Vivo
Source: Cancers (Basel). 2020 Jun 14;12(6):1570. doi: 10.3390/cancers12061570 (PMC7352231; doi:10.3390/cancers12061570)
Supplement: Supplementary file 1 [file cancers-12-01570-s001.pdf]

Supplementary Materials: Src Inhibitors Pyrazolo[3,4-d]pyrimidines, Si306 and Pro-Si306, Inhibit Focal Adhesion Kinase and Suppress Human Glioblastoma Invasion In Vitro and In Vivo

Marija Nešović, Aleksandra Divac Rankov, Ana Podolski-Renić, Igor Nikolić, Goran Tasić, Arianna Mancini, Silvia Schenone, Milica Pešić and Jelena Dinić

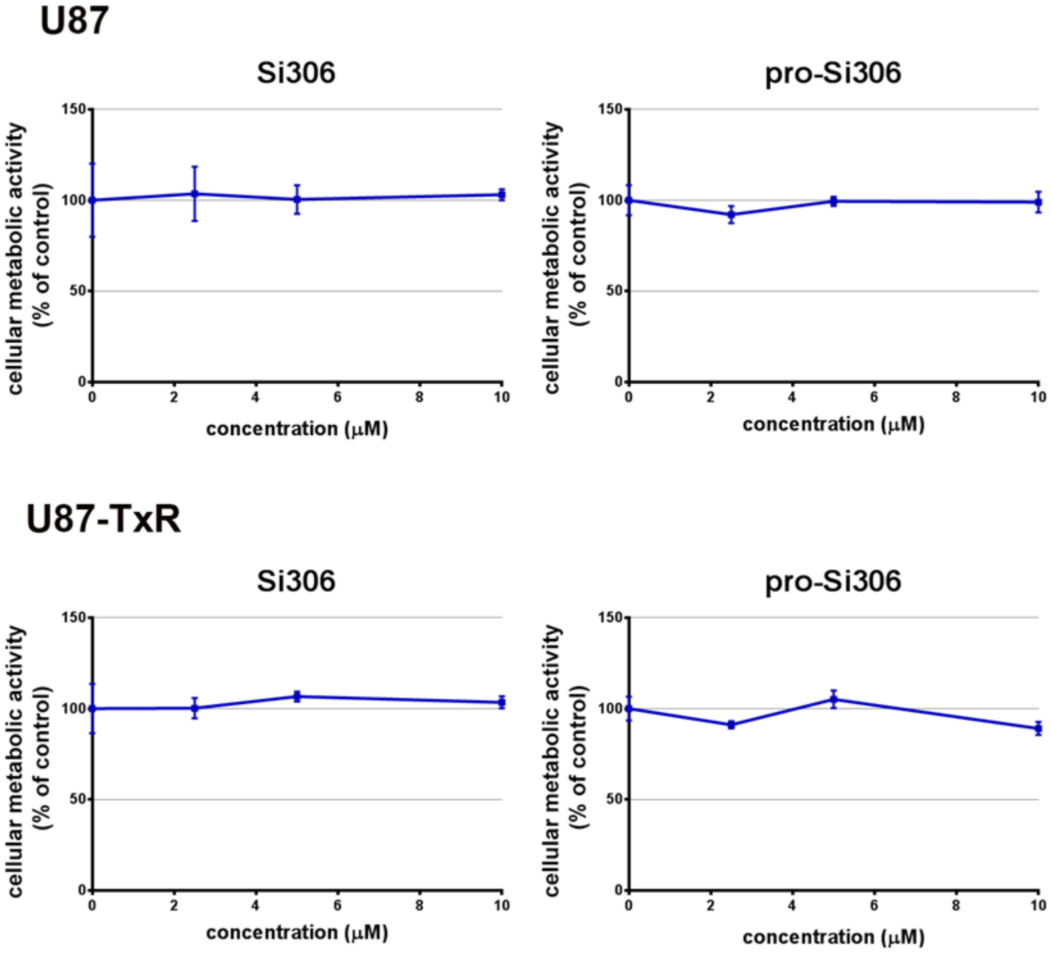

Figure S1. Sensitivity of U87 and U87-TxR cell lines to Si306 and pro-Si306 after 24 h treatment. Cellular metabolic activity was determined by MTT assay. Values are expressed as mean ± SD ($n = 3$).

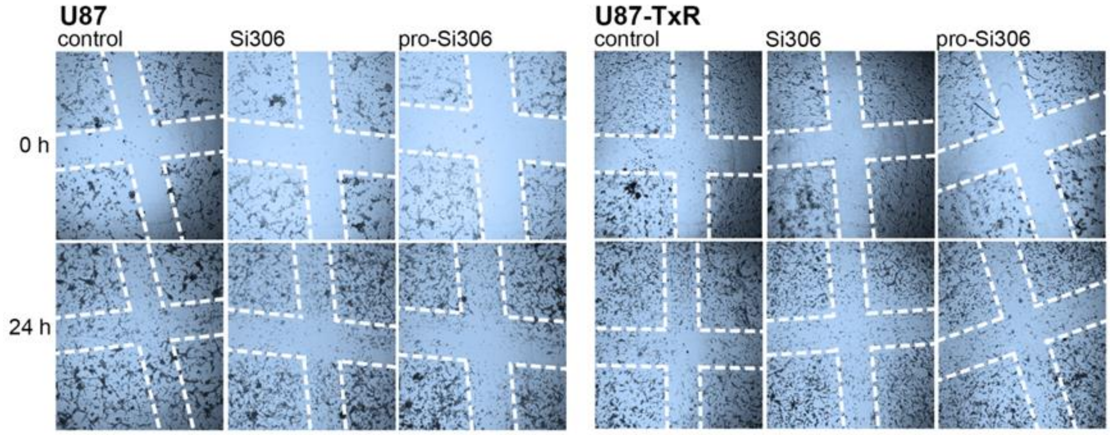

(**a**)

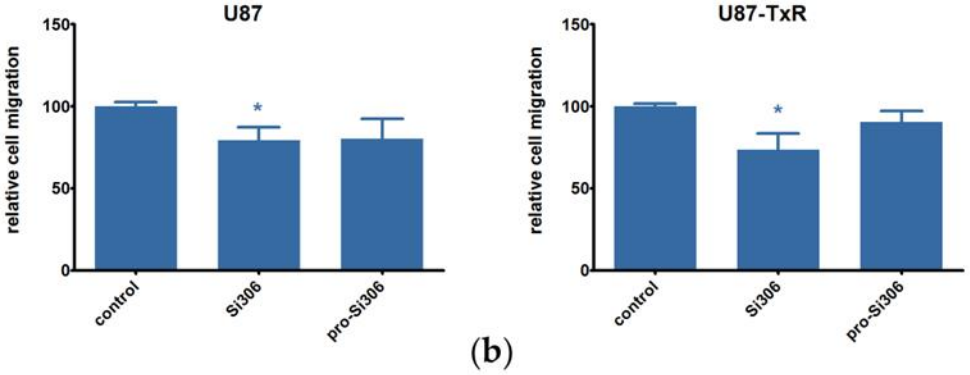

(**b**)

Figure S2. Anti-migratory effect of Si306 and pro-Si306 in GBM cell lines. (**a**) Representative images of wound healing in U87 and U87-TxR cells after 24 h treatment with 5 μM Si306 and pro-Si306. (**b**) Relative migration of U87 and U87-TxR cells. Values are expressed as mean ± SEM (n = 3). Statistical significance between treated and control group is shown as * ($p < 0.05$).

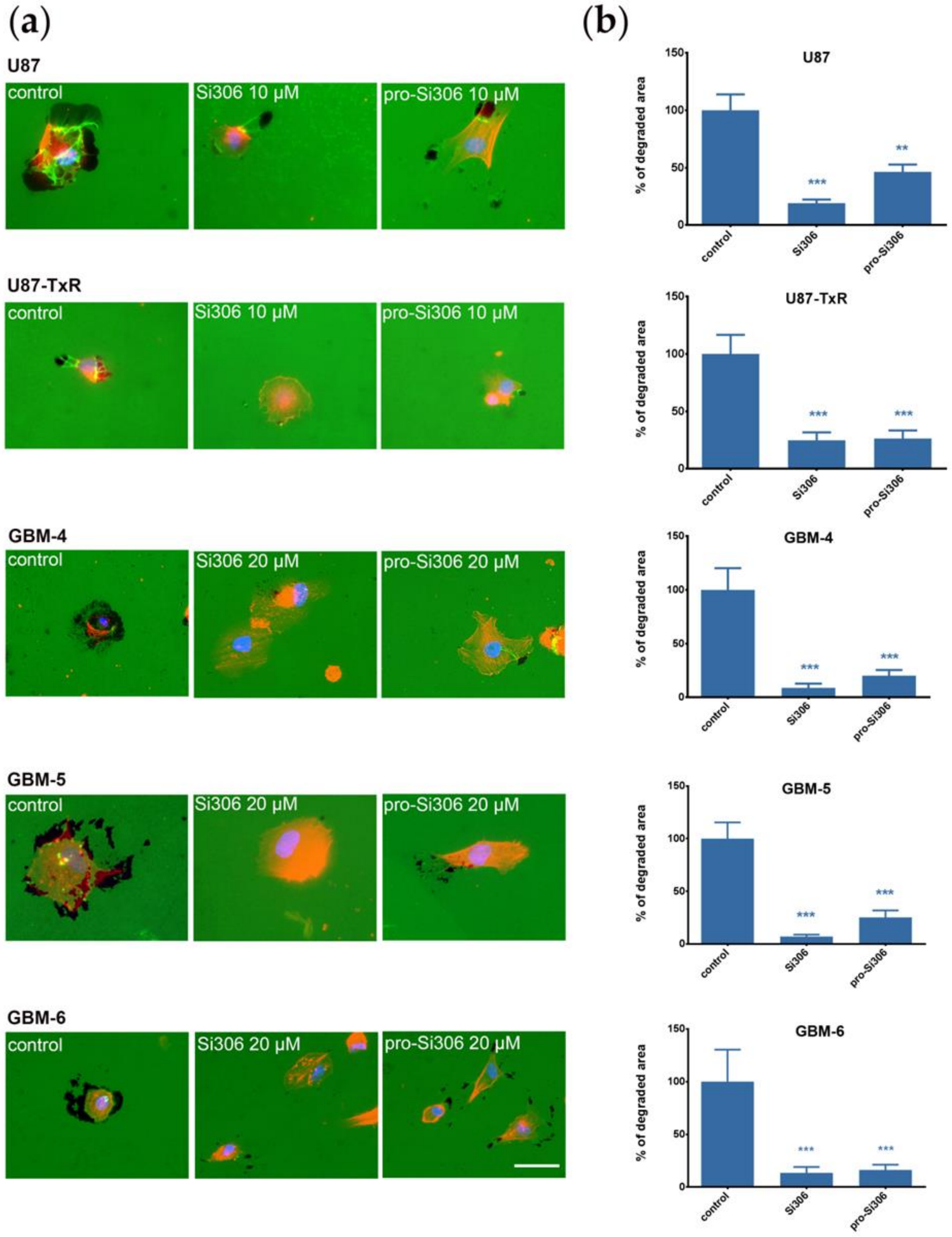

Figure S3. Si306 and pro-Si306 decrease the ability of GBM cells to degrade the ECM. (**a**) Representative images of gelatin degradation by U87 and U87-TxR cells treated with 10 μM Si306 and pro-Si306 for 24 h, as well as GBM-4, GBM-5, and GBM-6 primary cultures treated with 20 μM Si306 and pro-Si306 for 24 h. Scale bar = 30 μm. (**b**) Percentage of area degraded by U87, U87-TxR, GBM-4, GBM-5, and GBM-6 cells. Values are expressed as mean ± SEM ($n = 3$). Statistical significance between treated and control group is shown as ** ($p < 0.01$) and *** ($p < 0.001$).

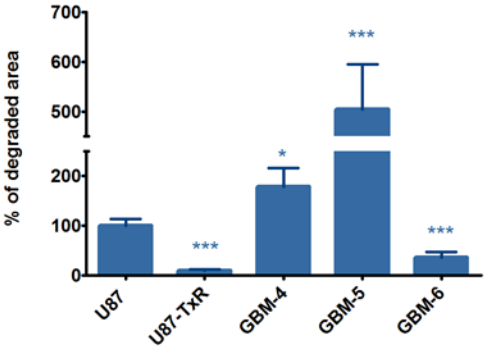

(**a**)

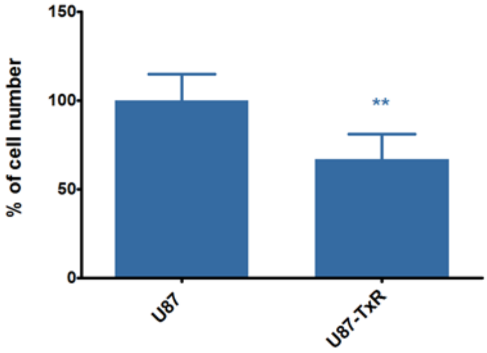

(**b**)

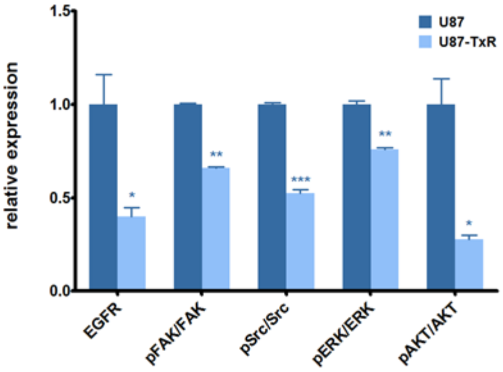

(**c**)

Figure S4. Invasive potential of GBM cells. (**a**) The ability of primary GBM cultures to degrade gelatin compared to U87 and U87-TxR cell lines expressed as the percentage of degraded area. (**b**) Invasive ability of U87 and U87-TxR cells expressed percentage of cells that invaded through matrigel and passed the membrane in Transwell assay. (**c**) U87 and U87-TxR cells' western blot data expressed as phosphoprotein level relative to total protein level (FAK, Src, ERK, AKT) or total protein expression (EGFR), all normalized to β-tubulin. All values are expressed as mean ± SEM (n = 3). Statistical significance is shown as * ($p < 0.05$), ** ($p < 0.01$), and *** ($p < 0.001$).

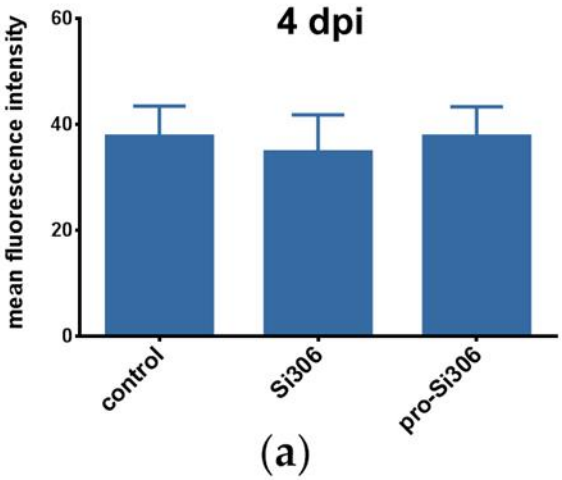

(a)

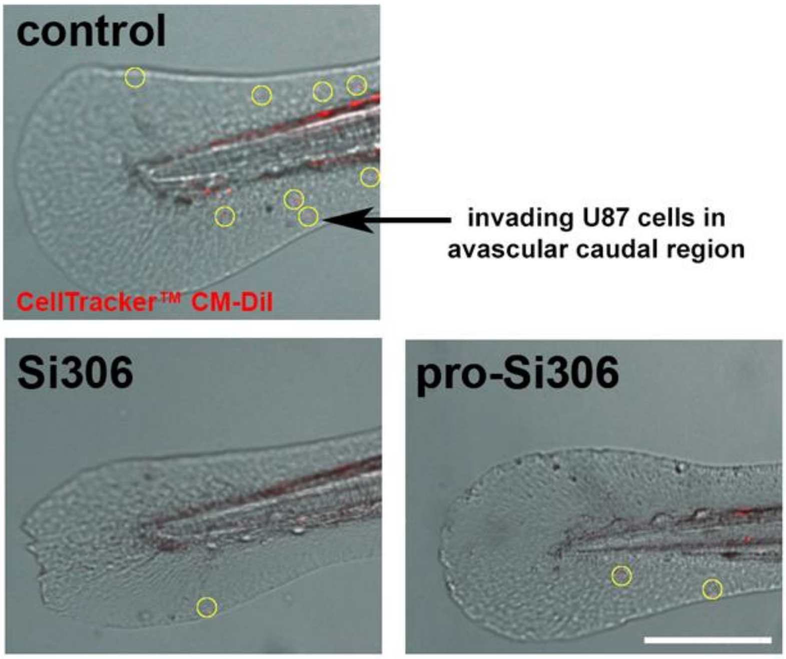

(b)

Figure S5. The effects of Si306 and pro-Si306 on GBM xenografts cell loss and invasion. (**a**) Mean fluorescence intensity of CM-Dil-labeled U87 cells in 4 dpi zebrafish embryos treated with 2.5 μM Si306 and pro-Si306 for 72 h. The steady CM-Dil fluorescence in treated groups indicates no cell loss compared to control. Values are expressed as mean ± SD. (**b**) U87 cells invade into the avascular caudal region of zebrafish embryos. Representative images of U87 xenografts in zebrafish embryos at 4 dpi are shown. Arrow and yellow circles indicate U87 cells that migrated out of the vasculature and into the avascular caudal region. The number of invading U87 cells in the tail region of xenografted embryos was notably reduced after 72 h treatment with 2.5 μM Si306 and pro-Si306. Scale bar = 500 μm.

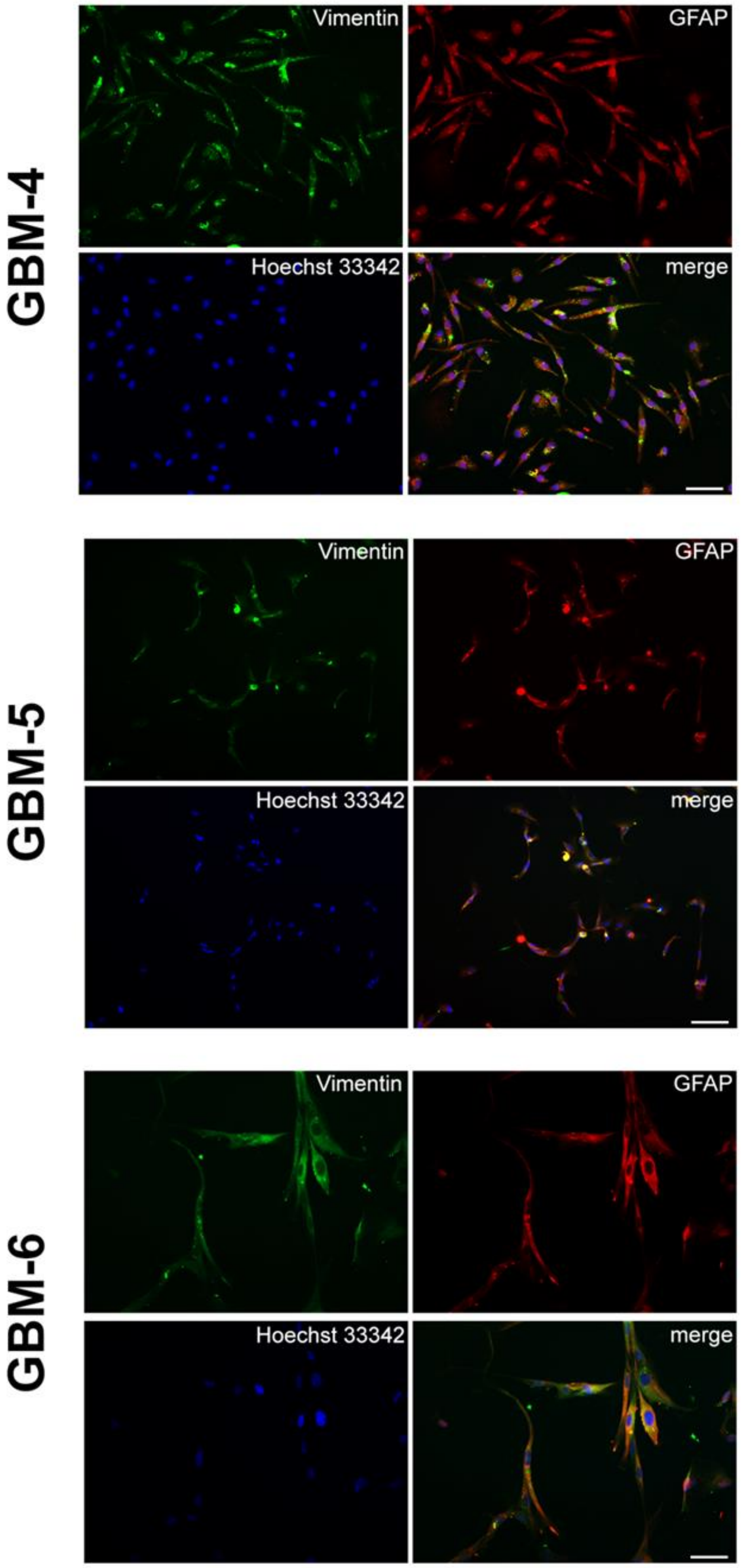

Figure S6. Characterization of primary GBM cultures. Representative images of immunostaining for GBM markers vimentin and GFAP in primary GBM-4, GBM-5, and GBM-6 cells. Scale bar = 50 μm.

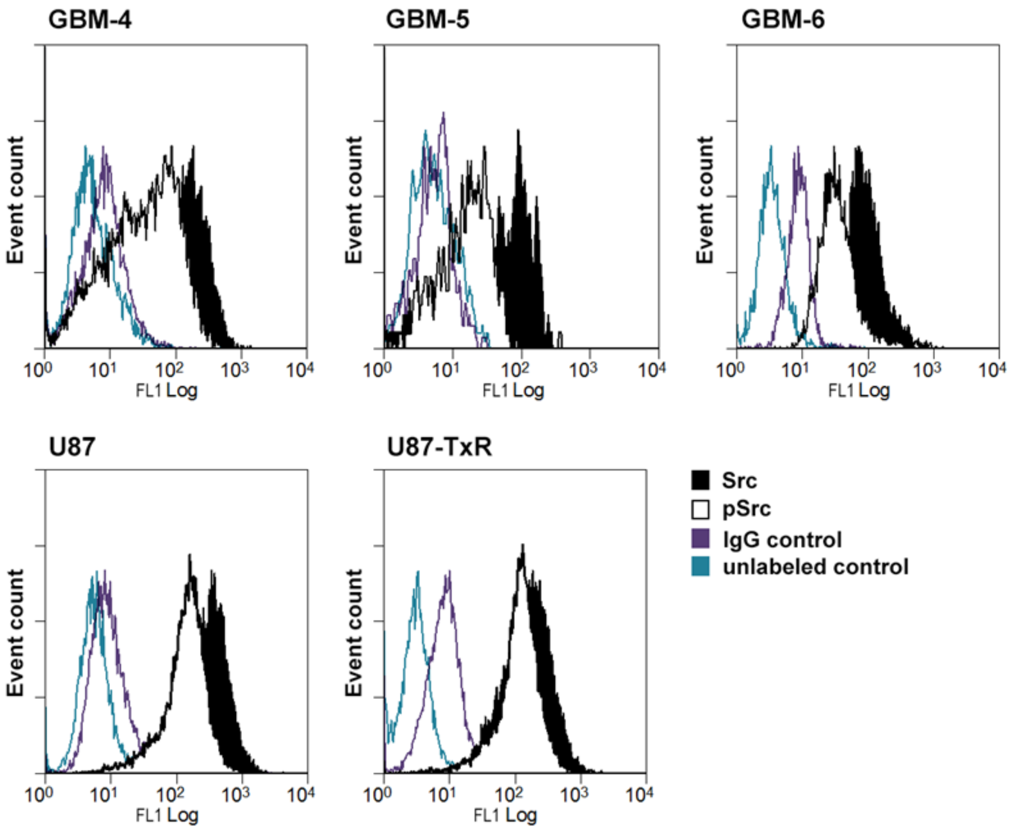

Figure S7. Flow cytometry controls for immunolabeled GBM cells. Flow-cytometric profiles representing signals obtained from cells labeled with rabbit anti-Src antibody or anti-pSrc antibody coupled with Alexa Fluor 488 anti-rabbit IgG(H+L), cells labeled with rabbit IgG XP® antibody coupled with Alexa Fluor 488 anti-rabbit IgG(H+L), and unlabeled cells.

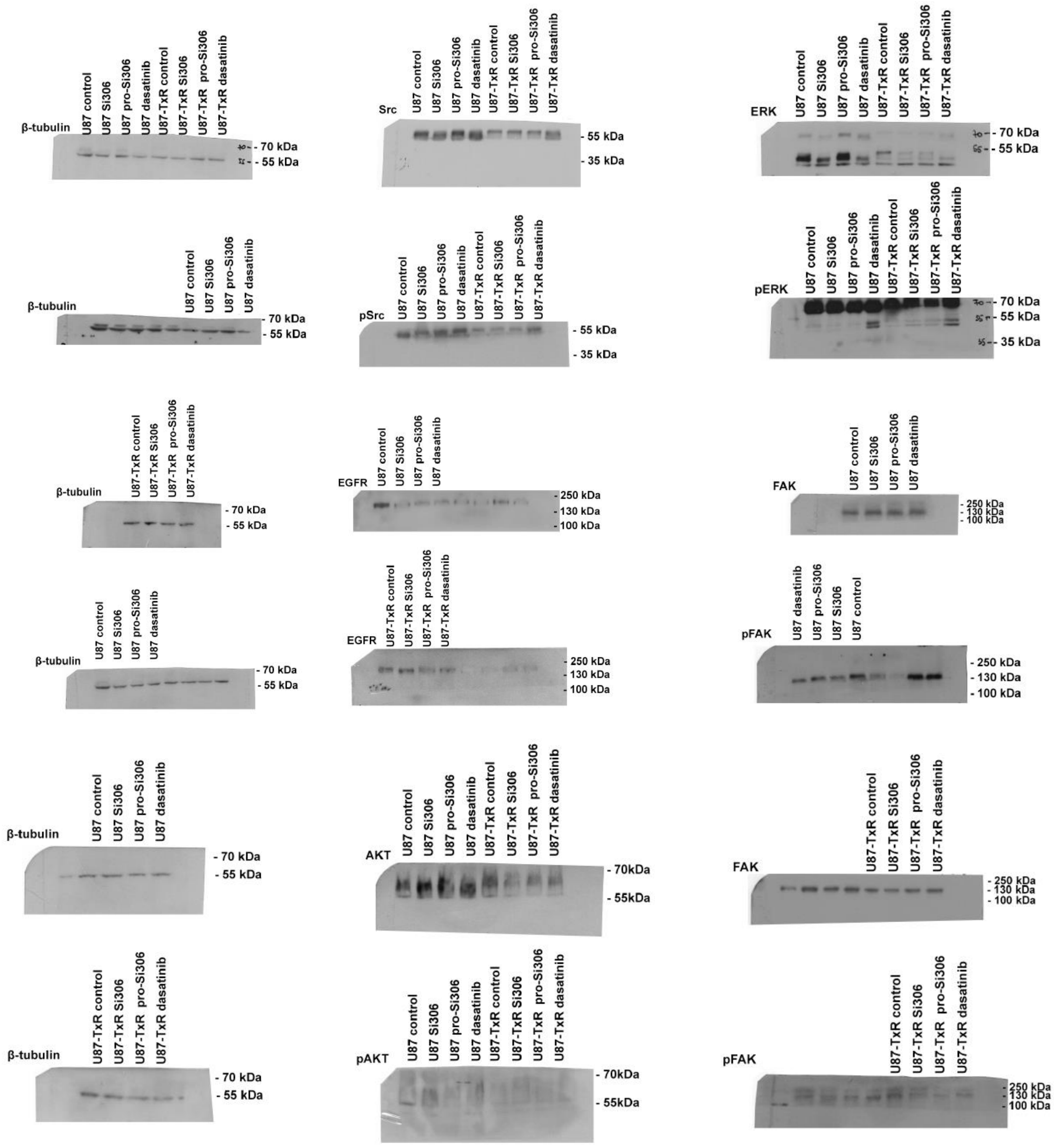

Figure S8. Western blots from Figure 5 and Figure 6 representing in Scr, pScr, EGFR, FAK, pFAK, ERK, pERK, AKT and pAKT in U87 and U87-Txr cells with 5 uM Si306, pro-Si306, and dasatinib for 24 h.

© 2020 by the authors. Submitted for possible open access publication under the terms and conditions of the Creative Commons Attribution (CC BY) license (http://creativecommons.org/licenses/by/4.0/).
